# Developing Regenerative Treatments for Developmental Defects, Injuries, and Diseases Using Extracellular Matrix Collagen-Targeting Peptides

**DOI:** 10.3390/ijms20174072

**Published:** 2019-08-21

**Authors:** Leora Goldbloom-Helzner, Dake Hao, Aijun Wang

**Affiliations:** 1 Surgical Bioengineering Laboratory, Department of Surgery, School of Medicine, University of California Davis, Sacramento, CA 95817, USA; 2 Department of Biomedical Engineering, University of California Davis, Davis, CA 95616, USA; 3 Institute for Pediatric Regenerative Medicine, Shriners Hospitals for Children, Sacramento, CA 95817, USA

**Keywords:** collagen, extracellular matrix, tissue engineering, regenerative medicine, collagen hybridizing peptide

## Abstract

Collagen is the most widespread extracellular matrix (ECM) protein in the body and is important in maintaining the functionality of organs and tissues. Studies have explored interventions using collagen-targeting tissue engineered techniques, using collagen hybridizing or collagen binding peptides, to target or treat dysregulated or injured collagen in developmental defects, injuries, and diseases. Researchers have used collagen-targeting peptides to deliver growth factors, drugs, and genetic materials, to develop bioactive surfaces, and to detect the distribution and status of collagen. All of these approaches have been used for various regenerative medicine applications, including neovascularization, wound healing, and tissue regeneration. In this review, we describe in depth the collagen-targeting approaches for regenerative therapeutics and compare the benefits of using the different molecules for various present and future applications.

## 1. Introduction

Collagen, the most abundant extracellular matrix (ECM) protein in mammals, regulates numerous bodily processes, from the molecular to the macroscopic scale [1]. It also plays a large role in dictating the overall mechanical and metabolic properties of tissues [1]. In many cases of developmental defects, injuries, and diseases, collagen is observed to be injured or dysregulated [2]. Therefore, collagen could be an ideal target for identifying and treating damaged sites. The study of native collagen in vivo has recently expanded to explore diagnostics and therapeutics in disease models heavily influenced by the abnormalities in collagen molecules and fibrils [3]. Molecules have been designed to bind directly, or hybridize, to the collagen strands making the targeting of these abnormalities much more feasible [4]. In this review, we further describe collagen’s structure-function relationship and its role in developmental defects, injury, and disease. Then, we introduce the collagen-targeting molecules used to date and the recent advances in their therapeutic and regenerative application.

## 2. Collagen in the Extracellular Matrix

### 2.1. Structure of Collagen

Collagen is a cell-responsive ECM protein that forms a tertiary structure capable of transferring signals and mechanical stresses to cells, while protecting them from fatal loads [5]. The collagen molecule is made up of three polypeptide chains intertwined to form a left-handed triple helical structure, via intra- and inter-chain hydrogen bonding. Each chain, termed an alpha chain, is made up of a repeating sequence of three amino acids—Glycine-X-Y. Glycine is necessary in the repeating amino acid sequence of collagen, due to its compact nature that enables the twisting of alpha chains into the triple helix structure. X and Y are amino acid placeholders, most frequently proline and hydroxyproline respectively, which dictate the mechanical and bioactive properties, and the exact steric effects of the triple helix structure [2]. The alpha chains, ~300 nm in length, undergo a modification process, including hydroxylation and glycosylation, to drive helix formation and transport, from the rough endoplasmic reticulum to the Golgi bodies, and finally outside the cell to become part of the ECM [2]. Collagen molecules are further arranged in a quasi-hexagonal crystal lattice and right-handed supertwist to form microfibrils [6]. Microfibrils aggregate and interdigitate through covalent cross-links to form a collagen fibril (ranging anywhere from 10–300 nm in diameter), which can be further aligned with other collagen fibrils to create a collagen fiber [7].

There are various collagen types that differ based on the possible combinations of alpha helices in the triple helix structure, which makes them tissue specific. To date, 27 types of collagen have been distinguished in humans [2,8]. For instance, type I collagen, the most common type of collagen occurring in ligaments, tendons, and muscles, is made up of two pro-alpha1 and one pro-alpha2 chains. These procollagen alpha chains are coded by *COL1A1* and *COL1A2* genes, respectively. Conversely, type II collagen is composed of three identical pro-alpha1 chains and occurs primarily in articular cartilage [9]. Therefore, engineering approaches meant to regenerate collagen containing tissue, need to account for the distinct biochemical and mechanical properties of the various types of collagen.

### 2.2. Biochemical and Mechanical Properties of Collagen

Although most tissues are composed of collagen, the diversity in type, amino acid sequence, and cell-protein or protein-protein interactions vary the biochemical and mechanical properties of each tissue, for optimal functionality. The subtle differences in properties affect the behaviors of cells in states of homeostasis and regeneration or repair. For many natural regenerative processes in the body, cells must be directed to repopulate at a defect site, recruit other cell types, and deposit ECM for organ function restoration, post-injury. Collagen participates in the sequestration of growth factors and other signaling molecules that aid in the paracrine regulation of cell behavior [10]. Several cell integrins bind to collagen molecules and sequester growth factors, causing a cascade of signaling pathways downstream to promote adhesion, proliferation, and differentiation [2].

Collagen is also heavily involved in mechanotransduction, affecting cell signaling pathways by changing its stiffness and the force applied directly to the cells. Collagen has high tensile strength, which allows it to transmit forces between tissues, while absorbing enough of a load to protect cells. Making up over 70% of the ECMs of skin, tendon, and cartilage, the strength, stiffness, and Young’s moduli of collagen molecules can significantly affect cell behavior and organ function [1,8]. Collagen fibrils are largely anisotropic due to the individual collagen molecules aligning along their longitudinal axis. This physical anisotropic nature of the molecules translates closely to a mechanical anisotropy of the overall collagen fibrils [11]. This property serves as a benefit in many tissues that require higher strengths and Young’s moduli (i.e., type I collagen’s moduli ranges from 5–11.5 GPa) in directions specific for biological functions [11]. Other proteins also possess collagen binding domains (CBD) that influence the cell behavior and physical properties of ECM. One example is glycosaminoglycans (GAGs), which are present in many tissues and closely associated with collagen. These GAGs are polysaccharides that have high negative charge densities and attract water to hydrate the tissues and absorb the forces applied. Overall, many properties of tissue-specific ECMs dictate their function and maintenance of a healthy phenotype, especially collagen’s biochemical and mechanical properties.

### 2.3. Role of Collagen in Developmental Defects, Injuries, and Diseases

Healthy collagen can positively promote migration, adhesion, and differentiation of cells to guide morphogenesis in development as well as to regenerate tissues at injury or disease sites [12,13]. However, collagen can also play an important role in developmental defects, injury, and disease. Collagen dysregulation can lead to its over- or underproduction, which can severely impact specific tissue functions. Excessive collagen remodeling, in diseases such as cancer or tissue fibrosis such as kidney, heart, or pulmonary fibrosis, leads to a stiffened ECM significantly restricting the elasticity in tissues that would normally need an elasticity factor, such as the lungs, muscles, and heart [14]. On the other hand, injured or degraded collagen can weaken tissue mechanical integrity and reduce its ability to sequester growth factors for differentiation or regenerative purposes, and respond to endogenous signals from cells for optimal mechanotransduction [2]. The origin of dysregulated ECM collagen can be hereditary, epigenetic, or environmental [15]. In all cases, understanding the role of collagen in developmental defects, injuries, and diseases can help establish improved targeting mechanisms designed for diagnostics, therapeutics, and regenerative purposes.

#### 2.3.1. Developmental Defects

Collagen undergoes extensive proteolytic remodeling and organization during development [16,17]. However, these processes can be stymied by genetic mutations that code for different collagen types, which lead to a wide range of congenital defects. These mutations affect the biosynthesis and processing of collagen from the amino acid sequence level to the tertiary structure level. Substitutions can result in the replacement of a codon for a critical amino acid (i.e., glycine), within the alpha chain, that prevents its propensity to form triple helices. Other deletions or insertions can affect collagen-processing enzymes, which cleave terminal regions of procollagen strands and can thus change the molecules’ solubility and/or properties. In developmental disease cases, such as osteogenesis imperfecta, mutations primarily in the *COL1A1* gene negatively impact the formation and folding of alpha chains present in type I collagen, causing its excessive accumulation [18,19]. This causes a downstream effect on triple helix formation and further processing that leads to a phenotype of brittle bones, which can be lethal perinatally, in the most severe cases. Mutations observed in *COL2A1* that stunt formation of alpha chains in type II collagen result in weakened mechanical properties and degraded cartilage tissue—a phenotype common in patients with chondrodysplasia [20]. Type IX collagen mutations can change the amino acids in the X or Y position to tryptophan, which is not naturally one of the amino acids in the sequence. Tryptophan does not allow for an ideal fit of alpha chains in the triple helix, which leads to dysregulation of collagen and developmental defects [21]. This mutation appears as intervertebral disc disease in many patients [2].

#### 2.3.2. Injuries

Environmental damage to collagen can take many forms. For instance, spinal cord injury is an acute injury that results in the high accumulation of fibrotic tissue and scar formation if untreated. Post-injury, a deposition of ECM meshwork, consisting primarily of type IV collagen, triggers inflammation and prevents the regeneration of axons at the lesion center both by physical blockage and by presentation of growth-inhibiting molecules [22]. This can cause any number of irreversible paralyses. Osteoarthritis is a progressive degenerative disease originating from an acute injury to the joint cartilage, which sets off type II collagen and general ECM breakdown. Breakage can occur on the order of collagen molecules in the unraveling of alpha chains, or on the order of collagen fibrils leaving accumulated collagen fragments at the injury site [2]. The degraded collagen at the injury site weakens its mechanical integrity. This leaves the injury site vulnerable to further damage and eventual lifelong disability [23]. Injuries of the anterior cruciate ligament also initiate rapid type I collagen turnover [24]. High enzymatic activity and severe collagen remodeling cause delayed recellularization and lowered mechanical properties of the regenerated tissue [25]. Collagen dysregulation takes a severe toll on tissues that mainly provide structural and mechanical stability, and acute injury that initiates this phenomenon can contribute to lifelong disability and chronic disease.

#### 2.3.3. Diseases

Disease can instigate the fibrosis or degradation of tissue which is primarily made up of ECM collagen. In the prevalent condition of atherosclerosis, fat and plaque buildup lead to thrombotic occlusion in vessel walls [26]. Not only is collagen damaged at the onset of this condition, but it is further damaged from the exposure to blood flow after a balloon angioplasty that corrects for the atherosclerosis and myocardial infarction (MI). Although cell binding sites allow collagen to aid in the regeneration of damaged tissues, exposed collagen in vessels can lead to a buildup of collagen-bound platelets causing the narrowing of vasculature and eventual thrombosis [27]. Cancers also possess a phenotype of dysregulated collagen often in the form of excessively remodeled ECM and fibrotic tissue [28]. Different cancers remodel various collagen types including medulloblastoma (type I collagen) [29], pancreatic cancer (type IV collagen) [30], epithelial ovarian cancer (type I and III collagen) [31], breast cancer (type I collagen) [28], and colorectal cancer (type VIII collagen) [32]. Though cardiovascular disease and cancer are two of the most common causes of death in the United States, other diseases, such as pulmonary, liver, and kidney fibrosis also suffer from dysregulation and chronic overproduction of collagen and other ECM proteins [4,33]. Tissue fibrosis often results in permanent scarring, organ malfunction and, ultimately death if untreated [4]. Due to its predominant role in ECM degradation, remodeling, and fibrosis, collagen provides the perfect target for new and widespread diagnostic, therapeutic, and regenerative technologies. To date, several molecules have been identified to specifically target collagen for diagnostic and therapeutic purposes. The subsequent sections further describe these molecules and their potential use in treatment of the above diseases in the form of tissue regeneration.

## 3. Collagen-Targeting Molecules

Targeting collagen has a widespread potential in understanding, diagnosing, and treating developmental defects, injuries, and diseases. We will discuss the various peptides derived from collagen binding domains, the antibodies designed to bind collagens at different statuses, and the collagen mimetic peptides that hybridize to the denatured collagen strands. The peptides range from 7–30 amino acids in length and target many types of collagen [4]. They have been primarily used in conjunction with growth factors and drugs to promote regeneration of blood vessels, bone, and cartilage. These functionalizations are detailed in subsequent sections.

### 3.1. Collagen Binding Peptides

#### 3.1.1. SILY

The peptide, RRANAALKAGELYKCILY, is derived from the platelet receptor that binds to α1 chains in collagen. The original molecule was discovered through the purification of platelet membrane receptors and competitive binding assays to α1 chains and type I fibrillar collagen [34]. More recently, the peptide has been altered at its internal cysteine, replacing the thiol group for cysteine with a hydroxyl group for serine [34]. Although the new molecule, RRANAALKAGELYKSILYGC (Kd = 0.86 µM), abbreviated to SILY, has a slightly lower binding affinity to type I collagen than its original counterpart, it has enabled the chemical conjugation of SILY to other molecules, primarily a large dermatan sulfate (DS) molecule [34]. Even though SILY has been noted with relatively limited binding specificity to collagen (personal communication with researchers in the field), its high binding affinity to collagen has led to extensive medical applications. Specifically, DS-SILY acts as a biomimetic of decorin, a natural proteoglycan that consists of a glycosaminoglycan (GAG) chain and a protein core, rich in leucine repeats that aid in the binding to type I collagen fibrils [34,35]. In cases of vessel injury or balloon angioplasty, endothelialization can be damaged and collagen exposed. This can lead to the buildup of collagen-bound platelets and restenosis of vessels [36]. The conjugated molecule competitively binds to the platelet binding sites, which reduces the potential for thrombosis [37]. DS-SILY has been shown to inhibit platelet accumulation and matrix metalloproteinase (MMP)-mediated collagen degradation as well as to reduce the effects of pro-inflammatory and pro-fibrotic factors, platelet-derived growth factor (PDGF) and interferon-γ (IFN-γ), which typically activate smooth muscle cell proliferation and migration to cause intimal hyperplasia [35,37,38,39,40].

#### 3.1.2. TKKTLRT

TKKTLRT is a peptide derived from the CBD in the collagen-degrading enzyme, collagenase [4]. The molecule was constructed from the complementary nucleotide sequence on interstitial collagen that codes for the region cleaved by the enzyme. Original reasons for this peptide’s synthesis were not for targeting collagen, but for inhibiting collagenase activity [41]. Collagenase can negatively impact the native ECM by breaking down collagen to cause excessive remodeling (e.g., cancer) or progressive degradation (e.g., osteoarthritis) in vivo [42,43]. Tissue inhibitors of metalloproteinase (TIMP) have been discovered to bind directly to collagenase molecules and prevent further cancer metastasis as ECM remodeling is a common phenotype of cancer because it enables cancer cell migration [43]. However, these TIMP molecules only target collagenase therefore reducing their applicability for regenerative use in sites of damaged tissue. TKKTLRT not only competitively binds to type I collagen with high specificity, which decreases the likelihood of collagenase-based degradation, but can be functionalized to deliver factors to stimulate regeneration of tissue in areas of dysregulation. To date, TKKTLRT has been conjugated to several growth factors and drugs to facilitate diabetic wound healing, neurogenesis, vascularization, and cellularization [27,44,45,46,47,48]. Subsequent sections will detail the specific nature of these functionalizations to promote tissue regeneration.

#### 3.1.3. WREPSFMALS

WREPSFMALS is a peptide derived from the von Willeband’s factor (vWF), which is an adhesive glycoprotein found in plasma, platelets, and endothelial cells [49]. vWF is heavily involved in clotting processes, interacting closely with factor VIII to control wound healing [50]. In cases similar to the previously described vessel damage due to myocardial infarction and balloon angioplasty, platelets can aggregate where collagen is exposed [36]. WREPSFMALS competitively binds to collagen, which mediates platelet adhesion and clotting, and reduces the likelihood of thrombosis [49,50]. This decapeptide primarily targets type I collagen but has also been shown to bind other types of collagen (e.g., type II) [49]. It binds to intact collagen and denatured collagen (i.e., gelatin) with lower binding affinities (Kd = 100 µM) and specificities than TKKTLRT (Kd < 100 µM) [44,45]. To date, WREPSFMALS has been used to improve vascularization and cellularization. It has also been conjugated to growth factors to improve diabetic wound healing and regeneration of the intestine [45,51]. Subsequent sections further describe the strategies used in conjunction with this peptide to promote tissue regeneration.

### 3.2. Collagen-Targeting Antibodies

Collagen-labeling antibodies have been designed to specifically target denatured collagen and generally inhibit tumor growth, to prevent further degenerative progression of cancer [52,53]. These monoclonal antibodies provide an advantage over collagen-binding peptides because the peptide molecules have a limited propensity to bind denatured collagen over intact collagen. For conditions where collagen molecules are denatured or excessively remodeled (e.g., osteoarthritis, cancer, etc.), these collagen-binding peptides fall short in their binding affinities. While these antibodies have a superior targeting specificity, they are limited in their sensitivity to certain types of collagen and methods of collagen denaturation (i.e., thermal, enzymatic, physical, etc.) [4]. Antibodies mAb HUIV26 and D93 (Kd = 6.5 µM) both target cryptic epitopes on type IV collagen that are exposed by the denaturation of collagen. Monoclonal antibody, mAb HUIV26, targets thermally denatured collagen to inhibit tumor growth whereas D93 can target both thermally and proteolytically (i.e., MMP) denatured collagen to inhibit tumor growth [52,53]. Since developmental defects, injuries and diseases undergo different modes of collagen denaturation, antibodies may not have as wide a range of applications in targeting collagen for tissue engineering.

### 3.3. Collagen Hybridizing Peptides and Collagen Mimetic Peptides

To address the disadvantages of previously described collagen-binding peptides and antibodies, researchers have developed a collagen mimetic peptide capable of hybridizing to any individual alpha chain that has unraveled from the normal tightly bound triple helix structure of collagen [54]. This molecule offers a technology that has specificity to denatured collagen and is broadly applicable for different collagen types and denaturation processes.

Collagen hybridizing peptide (CHP) is comprised of the repeating amino acids glycine, proline, and hydroxyproline, which match native collagen’s main repeating amino acid sequence and allow for strong and highly specific hybridization to denatured collagen strands. Formally known as collagen mimetic peptide (CMP), which ranges from 6–10 amino acid sequence repeats, CHP has been standardized to represent (Gly-Pro-Hydroxypro)_9_. Other forms of CMP have substituted hydroxyproline for another proline, but it has since been shown that hydroxyproline increases the stability of the triple helix structure by enabling the formation of additional stabilizing hydrogen bonds [55]. The high propensity to form triple helices gives the CHP molecule an advantage in locating areas of protein dysregulation and detecting diseases in the early stages. CHP offers an additional advantage to other collagen binding peptides by binding specifically to the denatured collagen molecules, in preference to intact collagen.

CHP’s tendency to assemble into triple helices makes it naturally self-assemble in solution, prior to use, which keeps it from hybridizing to the desired collagen. To resolve this issue, three main treatments have been developed. CHP’s binding affinities are heat sensitive so one method is to heat up the solution and then quench to room temperature before application to a tissue [56]. Another method involves physically attaching a photocleavable nitrobenzyl group to a center glycine in the CHP molecule to create bulky steric effects and prevent self-assembly. The application of UV light cleaves the caged CHP molecule to enable collagen-targeting [57]. Most recently, a third method utilizes a similar physical addition to the CHP molecule to hinder self-assembly and create steric repulsion between injected molecules. Each proline of the CHP molecule was replaced with a (2S,4S)-4-fluoroproline (f) residue. This altered the previously stable steric effects of proline and neighboring hydroxyproline residues of CHP molecules while maintaining hybridization capabilities with natural collagen chains that are not as rich in hydroxyproline (occurring only in 34% of Gly-X-Y triplets) [58,59].

At its inception, CHP was a functional diagnostic marker of disease and injury sites and a method for studying embryonic development and aging. It was conjugated to a fluorescent marker (carboxyfluorescein) to reveal both denatured and highly remodeled collagen in diseases such as osteoarthritis, myocardial infarction, glomerulonephritis, pulmonary fibrosis, and Marfan syndrome [4,17,57]. The most recent alteration of CHP that exchanges proline with (2S,4S)-4-fluoroproline (f) residues has also provided another mechanism by which to diagnose collagen dysregulation through fluorescent imaging [58]. CHP also has the potential to increase the efficiency of regenerative factor delivery, to optimize localization and minimize non-specific binding. CHP also binds to all types of collagen, as opposed to collagen-binding peptides that target a particular collagen type. While other collagen-binding peptides may possess a type-specific binding domain, CHP hybridizes to any triple helix structure of collagen molecules making it a peptide that can be widely used for different diseases, injuries, and congenital defects [17]. However, since CHP can bind to any denatured triple helix structure, it would not have the specificity to hybridize to certain denatured collagen types. In the case of spinal cord injury, delivering signals to aid in the regeneration of vasculature, muscle, bone and neural tissues would require a multitude of signals. For this reason, the type-sensitive collagen-targeting molecules may provide a competitive advantage to CHP in certain cases.

## 4. Strategies for Targeting Collagen in Regenerative Medicine and Tissue Engineering

### 4.1. Delivery of Biologic Drugs/Growth Factors

Collagen-rich ECM functions as more than just the mechanical structure supporting cells in vivo. The ECM interacts with cells in various ways, through binding domains and sequestered growth factors. As a framework made up of a diverse set of proteins, there are many opportunities for cells to bind to different binding domains, take up sequestered growth factors or immobilized drugs, and respond to mechanical cues prompted by the stiffness, stress relaxation or other mechanical properties of ECM. The advantage of delivering exogenous growth factors to diseased or injury sites is that they mimic the bioactive cues normally supplied by healthy ECM to sequester growth factors and control mechanical, biochemical, and metabolic properties of tissue. Approaches that provide these signals fall under the term developmental tissue engineering, where it is believed that restoring the endogenous signals to match growth factors sequestered by healthy ECM will aid in the full regeneration of tissue. Delivery of those cues as well as biologic drugs, however, have so far been non-specific and short-term. Collagen binding and hybridizing peptides have thus improved the affinity, specificity, and lifetime of growth factors and drugs binding to collagen [60]. In several studies, drugs and growth factors were conjugated to targeting molecules to drive angiogenesis, wound healing, and musculoskeletal tissue regeneration.

SILY has been commonly conjugated to DS to inhibit platelet accumulation in damaged blood vessels [37]. SILY has also been fused with growth factors to add a regenerative function to the collagen-binding peptide. Mussel adhesive protein (MAP), derived from the marine mussel, has special adhesive properties that aid in scarless skin regeneration [61]. It has been fused to SILY to target the overproduction of collagen present in scars. CBD-MAP was tested in a wound healing assay and surgical procedure where it showed promise in serving as collagen-targeting glue [62]. SILY has also been conjugated to nanoparticles (NP) loaded with peptide KAFAK, designed to inhibit mitogen-activated protein kinase activated protein kinase 2 (MK2) activity effectively reducing pro-inflammatory cytokine levels. This complex of peptides and particles were able to modulate the platelet accumulation and immune response commonly seen after blood vessel damage [63].

TKKTLRT has been used to provide a targeting functionality to several growth factors and drugs to promote angiogenesis, wound healing, and bone regeneration [27,44,45,46]. TKKTLRT was fused with vascular endothelial growth factor (VEGF_165_) to add a collagen-targeting property to the growth factor. The fusion peptide (CBD-VEGF) was then immobilized onto a collagen hydrogel and implanted subcutaneously in a mouse model to demonstrate improved vascularization [27]. The pair was then tested, through intracardiac injection, into the right ventricle of a mouse post-MI. Improved cardiac function was observed when compared to saline and plain VEGF injections, based on the low percentage of scar tissue and increased wall thickness in the infarct area [27,45]. Further applications with this collagen-targeting VEGF include implantation of a functionalized collagen hydrogel in a diabetic mouse model, demonstrating a higher wound healing rate, better vascularization, and a higher level of VEGF in the granulation tissue wound [46]. Fusion of TKKTLRT with stromal cell-derived factor-1α (SDF-1α) in another study of MI observed that the recombinant chemokine improved recruitment of stem cells to the ischemic heart promoting healthier cardiac function [34]. Several other growth factors have been fused with TKKTLRT including bFGF and BMP-2 for wound healing (e.g., uterine horn reconstruction, bladder regeneration, etc.) and mineralized bone matrix regeneration, respectively [45]. Dai et al. created a fusion molecule between CBD (TKKTLRT) and a fragment of cetuximab, an anti-epidermal growth factor receptor (EGFR) antibody [47]. These studies allowed for the sustained release of the drug from a collagen scaffold for improved endogenous neurogenesis for acute spinal cord injury [48]. WREPSFMALS has also been fused with similar growth factors to TKKTLRT, but targets a different binding site on collagen and binds with a lower affinity. These growth factors include bFGF, BMP-2 and EGF to regenerate many tissues for diabetic wounds and mature bone (with trabeculae, and medullar cavities and blood vessels) [45,51].

### 4.2. Delivery of Peptides

A different collagen-targeting approach to tissue regeneration and immobilization of growth factors has been shown using CHP molecules conjugated to various peptides. CHP was attached to a pro-angiogenic cell-binding domain of VEGF termed QK. The CHP-QK molecules efficiently hybridized to hydrogels with varying degrees of denatured collagen (i.e., gelatin) and to poly (ethylene glycol) diacrylate hydrogels functionalized with other CHP molecules [64]. Substance P (Sub P) is a peptide that binds to the nerve receptor neurokinin and regulates vasodilation, angiogenesis, and immune responses. Sub P has also been shown to improve the proliferation of fibroblasts and epithelial tissue, which demonstrates its application for wound healing. Sub P was added to CHP to improve targeted wound healing of damaged collagen [65]. In these studies, endothelialization and neovascularization were achieved more efficiently in sites of degraded collagen and posed a competitive approach to presenting soluble growth factors to diseased tissue [64,66]. It also allows the delivery method to be modified from a local injection to an intravenous injection since CHP would not hybridize to any intact collagen on the way to the injury site. Tetsuji Yamaoka’s research lab has also used the collagen hybridizing sequence modified with integrin α4β1 ligand, REDV (POG7G3REDV) for vascular graft application [67,68]. This study showed that integrin-mimicking peptides have the potential to recruit host cells for many purposes including re-endothelialization of grafts [67].

Functionalization of the collagen-targeting molecules is still in the early stages of its application for regenerative purposes (Table 1). Immobilizing drugs, growth factors, and peptides to scaffolds and damaged sites in the body holds much potential in the field of regenerative medicine. Collagen binding and hybridizing molecules are advantageous in their use when combined with regenerative factors. There is promise in expanding the pool of molecules used and improving the collagen-targeting strategies for specific regenerative applications.

### 4.3. Development of Bioactive Surfaces

The application of tissue engineering approaches to regenerative medicine often involves the use of biomaterial hydrogels and scaffolds, made with either naturally sourced or synthetic materials [69]. One of the benefits of naturally derived constructs is that they contain at least some inherent cell-interactive properties, but they fall short in studies that require an independent control of properties (e.g., decoupling mechanical and biochemical properties) [70]. Synthetically derived scaffolds have the advantage of having independently tunable properties, but remain inert to their host cells and prevent the natural integration with the host tissue. Because the synthetic biomaterial can sometimes be hydrophobic, the scaffold risks high levels of surface protein adsorption. These hydrophobic surfaces can expose platelet binding regions on the adsorbed proteins, causing unwanted clotting and eventual fibrous encapsulation that naturally occurs when the body’s immune system senses foreign material [71]. Ideally, tissue engineered constructs should be tunable in every property so that scaffolds can be appropriately designed for various applications, but would also possess the ability to integrate with the host tissue and trigger minimal immune responses [72]. Several collagen-targeting molecules, specifically CHP, have been conjugated to otherwise inert biomaterials for these regenerative purposes (Table 2).

Studies have explored functionalizing synthetic scaffolds with CHP to aid in host ECM integration and to promote cell adhesion through ECM deposition. In conjugating CHP to poly (ethylene glycol) diacrylate hydrogels, researchers could drive capillary formation using the CHP-modified VEGF-mimetic peptide, CHP-QK [64]. CHP’s propensity to hybridize to other CHP strands allowed the mimicking of collagen molecules to target synthetic implants. Angiogenesis was improved in the burn wounds of a mouse model when tunable synthetic biomaterials had immobilized bioactive CHPs [66]. Poly (ethylene oxide) diacrylate (PEODA) hydrogels and PV membranes were also functionalized with CHP to better differentiate mesenchymal stromal cells (MSCs) toward a chondrogenic phenotype, and thus improve chondrogenesis. It was presumed that the functionalization allowed cell deposition of collagen and associated GAGs for improved mechanical support-an essential aspect of the chondrocytes’ microenvironment [73,74]. CHP was also used to create gradients both spatially and temporally on surfaces to drive regenerative processes that rely heavily on gradients. Varying the chain length of CMPs changed the propensity to form triple helix and therefore affected CMPs’ affinities to bind collagen film [75]. These techniques built off previous studies that functionalized biomaterials with drugs (e.g., drug-eluting stents) to provide a competitive aspect of spatial and temporal control—two parameters that hold important roles in general tissue regeneration.

### 4.4. Detection of Collagen Damage in Decellularized ECM Scaffolds

A common tissue engineered construct used for regenerative purposes is a decellularized ECM scaffold of desired tissues [76]. There are several benefits of using the natural ECM as a physical niche for regenerating diseased tissue. Natural ECMs match the exact protein makeup of healthy tissues in particular locations. They are also bioactive constructs containing cell binding domains, which drive cell adhesion, proliferation, differentiation, migration, and many other cell behaviors. Furthermore, the mechanical properties of natural ECMs allow transmission of precise mechanical cues to native cells for optimal function and regeneration [77].

Researchers have explored many decellularization techniques, with the intention of recellularizing and re-implanting ECMs in a diseased or injured site for regeneration [78,79,80]. These techniques can use physical, chemical, or enzymatic methods to detach and eliminate all cellular components prior to recellularization [81]. They also range in harshness, which can pose obstacles regarding the preservation of structural integrity, protein degradation, and loss of bioactive factors [82,83,84,85,86]. These cell-interactive and cell-responsive aspects of the decellularized ECM give it a competitive advantage over many other natural or synthetic engineered scaffolds so they cannot be sacrificed during the decellularization process itself [87]. Hwang et al. used CHP to assess damage to the ECM morphology after different chemical decellularization methods were performed [88]. A fluorescently tagged CHP molecule was used to label denatured collagen in porcine urinary bladders and porcine cruciate ligaments split into four experimental groups and treated with four detergents often used in decellularization methods (Triton X-100, SDS, SD, CHAPS). The authors found SDS to be harshest on the collagen molecular structure, followed by Triton X-100. While SD and CHAPS also appeared to alter ECM morphology, these groups were only affected on a macroscopic scale showing changes to collagen’s fibrillar structure. Both types of modifications to the ECM structure could have lasting effects on its mechanical integrity and bioactive features [88].

It is crucial to have a mechanism like CHP for observing the structure and dysregulation of collagen during technical procedures and for providing the best chance of success for regenerating tissues in vivo. Not only can this technique identify superior chemicals for decellularization, but it has the potential to be used in vivo to assess ECM turnover after collagen-based scaffolds are implanted [89].

### 4.5. Delivery of Genetic Materials

Drug and gene delivery methods have been used in attempts at reducing ECM-mediated fibrosis and the general overproduction of collagen. However, they have fallen short in their targeting ability thus far. The benefits of using gene delivery, rather than high doses of growth factors, are the sustained release of plasmids, efficient transfection due to local and specific delivery, and lower chance of tumor/cancer development [55,90]. Incorporating a collagen-targeting aspect to gene delivery may pose a competitive technique for delivering genetic materials, such as plasmids, to collagen rich tissues and treating diseases. Some chemical modifications of collagen can influence collagen’s biochemical and mechanical properties through the blocking of binding domains. Therefore, CMPs hold great benefits due to their hybridization to denatured collagen strands without chemically modifying the collagen molecules. Furthermore, the efficiency of covalent chemical binding is low, due to the complexity of collagen’s tertiary structure. CMPs were used to immobilize DNA polyplexes containing pMV-GLuc plasmid on collagen films and hydrogels. CMPs maintained a sustained release of plasmid for over 2 weeks—longer than the usual time scale of hours for genes to diffuse out of constructs [55]. Cells were able to take up genes with a higher transfection efficiency measured by a higher Caveolin-1 silencing effect reported in one study [90]. In this study, gene delivery using CMPs proved a competitive approach to delivering immobilized, or soluble, growth factors to engineered constructs and other regenerative techniques.

This technology is tailorable to regenerative applications and can improve the long-term regenerative effects on injured tissue. In other technologies developed for regenerative purposes, biomaterial implants may induce inflammatory responses and delivering growth factors may only have temporary effects. Gene therapy provides the unique ability to change natural processes to promote long-term regeneration effects and can be a cheaper and more stable approach over delivery of growth factors [90]. Although many genes have not been explored as targets for this collagen-targeting system, many have the potential to improve angiogenesis, wound healing, and treat hereditary and developmental diseases.

## 5. Conclusions and Future Therapeutic Applications of Collagen-Targeting Molecules

Collagens play a very significant role in the regulation of developing and maintaining healthy tissues. Collagens are a key component of the extracellular matrix in most tissues and can be a main identifier of diseased tissue when dysregulated. Though extensive research has worked on targeting collagen for regenerative and therapeutic efforts, this field has not been thoroughly exhausted in its applicability. Beginning as a method for diagnostics in fibrotic and degraded tissue diseases and a technology for studying early development, collagen-targeting strategies have expanded to therapeutic approaches such as delivering and immobilizing growth factors and genetic materials for disease treatment [4,45]. There are future promising opportunities to deliver a wide range of materials via collagen-binding or hybridizing peptides. Modifications of cell membranes, exosomes, drugs or specific cell-binding peptides with these targeting molecules can further drive and localize regeneration in a more thorough approach. Functionalizations can be used to further explore therapeutics for other diseases and developmental defects including atherosclerosis, myocardial infarction, pulmonary fibrosis, cirrhosis, and spina bifida [4]. Not only will collagen-targeting strategies be used for more regenerative applications, but there are also opportunities to better understand the fundamental mechanisms of developmental defects, injury, and disease, as well as to be potentially applied for personalized medicine and drug screening applications. With so many possible applications of these technologies, it is important to better understand the mechanisms and differences of these collagen-targeting molecules and the feasible functionalizations that could improve the effectiveness of tissue regeneration.

## Figures and Tables

**Table 1:** Functionalized collagen-targeting molecules for regenerative applications.

Collagen- Targeting Molecule	Target	Functional Group	Regenerative Application	Ref.
CHP	dn-Col	QK	Angiogenesis	[64,66]
		SubP	Wound healing	[65]
SILY	Type I Col	MAP	Scarless skin regeneration	[62]
		KAFAK-loaded NP	Anti-inflammatory regulation	[63]
TKKTLRT	Type I Col	VEGF	Neovascularization, cardiac repair post-MI	[27,45,46]
		bFGF	Neovascularization, uterine horn reconstruction, bladder regeneration, chondrogenesis	[45]
		BMP-2	Mineralized bone matrix regeneration	[45]
WREPSFMALS	Type I Col	EGF	Intestinal crypt regeneration	[45]
		bFGF	Diabetic wound healing	[51]
		BMP-2	Mature bone regeneration	[45]
POG7G3REDV (CMP_7_)	dn-Col	REDV	Endothelialization	[67,68]
mAb HUIV26	Thermally dn-Col IV	*n*/a	Inhibition of tumor growth	[53]
D93	Enzymatically dn-Col IV	*n*/a	Inhibition of tumor growth	[52]

**Table 2:** Biomaterial functionalization using collagen-targeting molecules for regenerative application.

Collagen-Targeting Molecules	Target	Functionalized Biomaterial	Regeneration Application	Ref.
CHP	dn-Col	PEGDA	Angiogenesis	[66]
		PV membranes	Chondrogenesis	[73]
		PEODA	Chondrogenesis	[74]
CMP_X_-PEGDA	dn-Col	Collagen film	Spatio-temporal bioactive factor release for tissue repair	[75]

## Abbreviations

ECM	Extracellular matrix
GAGs	Glycosaminoglycans
dn-Col	Denatured collagen
CBD	Collagen binding domain
MMP	Matrix metalloproteinase
MI	Myocardial infarction
DS	Dermatan sulfate
vWF	von Willeband’s factor
TIMP	Tissue inhibitors of metalloproteinase
CHP	Collagen hybridizing peptide
CMP	Collagen mimetic peptide
MAP	Mussel adhesive protein
NP	Nanoparticles
bFGF	Basic fibroblast growth factor
VEGF	Vascular endothelial growth factor
MK2	Mitogen-activated protein kinase activated protein kinase 2
SDF-1α	Stromal cell-derived factor-1α
QK	VEGF mimetic peptide
Sub P	Substance P
EGF	Epidermal growth factor
EGFR	Epidermal growth factor receptor
PEGDA	Poly(ethylene glycol) diacrylate
PV	Poly(vinyl alcohol)
PEODA	Poly(ethylene oxide) diacrylate
PLGA	Poly(lactide-co-glycolide)
MSC	Mesenchymal stromal cell
pMV-GLuc	Portal-mesenteric vein glucose
